# Spatial dynamics in health and disease: from neurodevelopment to therapeutic target identification for inflammatory diseases

**DOI:** 10.1038/s41392-026-02589-5

**Published:** 2026-02-09

**Authors:** Matteo Barberis, Jinkun Xie

**Affiliations:** 1 Molecular Systems Biology, School of Biosciences, Faculty of Health and Medical Sciences, University of Surrey, Guildford, Surrey UK; 2 Centre for Mathematical and Computational Biology, CMCB, University of Surrey, Guildford, Surrey UK; 3 Immunology, School of Biosciences, Faculty of Health and Medical Sciences, University of Surrey, Guildford, UK

**Keywords:** Predictive markers, Predictive medicine

In a recent study published in *Nature*, Zhang, Rubio Rodríguez-Kirby and colleagues accurately recorded temporal, dynamic changes during brain development revealing the program underlying chromatin accessibility of genes involved in myelination and axonogenesis.^1^ By developing an in situ spatial tri–omics platform – spatial ARP-seq and spatial CTRP-seq – at a near single-cell resolution that simultaneously profiles the genome-wide chromatin state, the whole-transcriptome RNA and the proteome, the study unravels the spatiotemporal dynamics of brain development and neuroinflammatory responses, with an atlas of the mouse postnatal brain benchmarked to corresponding regions of the developing human visual cortex.^1^

Brain development and demyelinating injury are regulated across layers of cellular information: chromatin accessibility and epigenetic modifications (e.g., H3K27me3 repression) shape transcriptional potential, while RNA and protein outputs can diverge, making the in situ tri–omics co-profiling essential for resolving the temporal scale. Leveraging this approach, Zhang et al. identified in the wild type cortex layer-defining transcription factors whose chromatin accessibility persists in time and spreads across layers even after their RNA expression declines, consistent with an “epigenetic trailing” of developmental states. In the corpus callosum, myelin genes were epigenetically primed before full transcriptional and protein-level myelination, with patterns organised across callosal subregions in coordination with layer-specific projection neuron tracts.^1^ Conversely, in a lysolecithin-induced focal white-matter demyelination mouse model, the spatial tri–omics platform reveals coordinated changes in chromatin accessibility, H3K27me3-associated silencing, RNA expression, and protein abundance for oligodendrocyte and myelin genes across de- and re-myelination, together with delayed microglial activation and neuroinflammatory responses in distal white-matter tracts. Within primary and distal lesion-like regions, microglia occupy distinct multimodal states with gene modules overlapping with known disease- and injury-associated programs, while the overall neuroinflammatory response follows similar spatial and temporal trajectories.^1^

While most spatial multi–omics studies employ integrated modalities that are measured in separate, matched tissue sections or in independent datasets – with only a subset of newer platforms co-profiling two modalities in the same section^2^ –, the study of Zhang et al. extends this integration to spatial tri–omics at near-cellular resolution, jointly mapping chromatin state, RNA, and proteins from the same slice, to unravel dynamic changes in the brain.^1^ The demyelination model underscores a central message: immune and inflammatory programs are inherently spatial and multi-layered, and are best understood when different –omics modalities are read out together in situ.^2^ Spatial tri–omics maps sampled across disease timepoints (stages) may therefore systematically reveal features whose chromatin opening, transcriptional induction, and protein upregulation follow distinct temporal orders. Thus, this approach may have significant implications for personalised medicine strategies aiming to provide a rational basis (i) to prioritise early versus late intervention targets, (ii) to identify biomarkers associated to disease onset and progression, and (iii) to deliver combinatorial therapeutic strategies.

However, the integrative methodology used in the study of Zhang et al. warrants further consideration. In this study, biological insights are mainly derived from modality-specific clustering and pairwise integration of RNA and ATAC (via SpatialGlue and related tools), joint spatiotemporal patterning of RNA and chromatin accessibility through negative binomial generalized additive model (NB-GAM), and cell–cell communication inferred from spatial RNA alone.^1^ This reflects the prevailing paradigm in many spatial multi–omics analyses, where integration proceeds by aligning selected modalities into a shared latent or spatial space and correlating their spatial or temporal patterns.^2^ In principle, such a pattern- and correlation-based integration is powerful, but it has the limitations to be largely correlative and to not fully capture mechanistic links across –omics layers. Moreover, the cell–cell communication analysis – through CellChat and NICHES – leverages only the transcriptome, even though additional chromatin and protein information is available.^1^ To move away beyond purely correlative multi–omics integration strategy, a knowledge-based network integration may be pursued: mapping genes, proteins, and metabolites onto curated biochemical pathways and networks (for example the KEGG database, the Reactome pathway database and related resources), and linking modalities along known biochemical and signalling interactions. Through this strategy, –omics data may be mapped on biochemical maps to identify for instance the metabolic routes underlying a disease condition.^3^ These pathway- and network-based representations allow spatial tri–omics readouts to be embedded in mechanistic, molecular circuits rather than only in abstract latent spaces. In tri–omics spatial datasets, such a mechanistically informed feature can then be coupled to predictive frameworks such as the recently proposed single-cell –omics-based Disease Predictor through AI (scDisPreAI),^4^ a unified framework that leverages AI to integrate single-cell –omics data. By doing so, it enables prediction of disease and disease stages as well as multi–omics biomarker discovery, while keeping the inferred biology interpretable at the level of individual genes, modules, and pathways.

Because existing pathway databases are incomplete and require time to incorporate new findings, large language models (LLMs) may be used to mine recent literature for additional putative links across –omics features, which would then be reconciled with curated networks and treated as hypothesis-generating rather than definitive. Applied systematically across different tissues and disease contexts, the integration of spatial–omics and knowledge-based network can prioritise novel targets and highlight convergent functional modules that may overlap with targets of existing drugs, thereby generating testable hypotheses for drug repurposing.

**Fig. 1:**
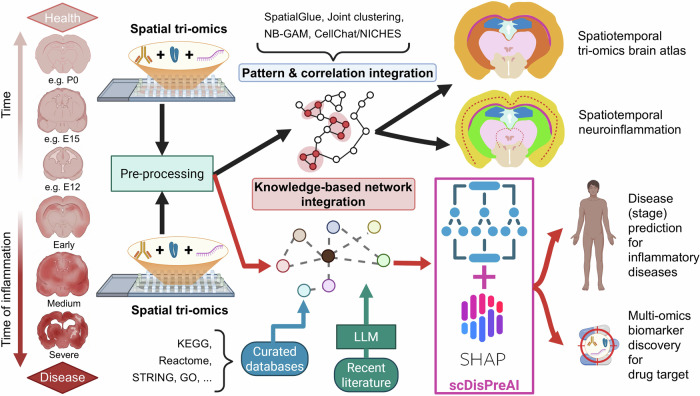
Spatial –omics workflow for building atlas of inflammatory dynamics and for disease prediction and biomarker discovery. (Left) DBiT-based spatial tri–omics (spatial ARP-seq: ATAC–RNA–protein; spatial CTRP-seq: H3K27me3 CUT&Tag–RNA–protein) is applied to mouse brains across postnatal development (top, health state) and to brains subjected to lysolecithin-induced demyelination at 5, 10, and 21 d.p.l. (bottom, disease state). In each section, chromatin state (ATAC or H3K27me3), transcriptomes and antibody-derived protein tags are captured from the same spatial pixels, followed by pre-processing: normalisation, batch correction, and quality control. Upper black arrows illustrate how Zhang et al. use these data primarily to build biological atlases: a spatiotemporal tri–omics brain atlas and maps of spatial dynamics during neuroinflammation. These are achieved through pattern- and correlation-based integration (SpatialGlue and joint RNA–ATAC clustering, negative binomial generalised additive model (NB-GAM) spatiotemporal modelling, and RNA-based cell–cell communication with CellChat and NICHES), with the protein layer (ADT and complementary CODEX imaging) used to refine cell types and validate spatial patterns. Lower red arrows highlight the proposed extension of spatial tri–omics for disease (stage) prediction and multi–omics biomarker discovery. Following the same pre-processing steps, multi–omics features from healthy and diseased tissues are integrated by (i) knowledge-based network integration, mapping genes, proteins and metabolites onto curated molecular interaction and pathway databases (e.g. KEGG, Reactome, STRING, Gene Ontology), and (ii) mining recent literature using large language models (LLMs), thereby linking modalities along known biochemical and signalling edges rather than by correlated patterns. These network-informed representations are then fed into an explainable AI framework such as the single–cell–omics–based Disease Predictor through AI (scDisPreAI), where predictive models plus SHAP-based interpretation support disease and disease-stage prediction and the prioritisation of spatially and mechanistically grounded multi–omics biomarkers as potential drug targets. Brain graphics were generated using Gemini. Created with and adapted from BioRender.com

Currently, no unified, disease-centric framework exists that can jointly perform multi–omics disease prediction, leverage spatial structure, build mechanistic networks, and generate testable biological hypotheses. To understand complex, inflammatory diseases, the innovative spatial tri–omics framework presented by Zhang et al. and the discussed emerging integrative strategies lay a solid foundation for multi–omics disease modelling, biomarker discovery, and target prioritisation (Fig. 1). Looking ahead, spatial multi–omics integration maps can provide time- and space-resolved readouts to mechanistically characterise the onset and progression of inflammatory (brain) diseases, such as for example the neuroinflammatory niche and glial-neuronal interactions observed within the amyotrophic lateral sclerosis (ALS) brain or spinal cord tissue sections. Notably, disease mechanisms modulated by spatial multi–omics biomarkers may be reflected in metabolic changes, with metabolites being detected in the blood,^5^ a viable biofluid to clinically unmask inflammatory diseases such as ALS. Complementarily, mechanistic networks that combine curated pathways with literature-mined relations that are linked to disease-prediction models offer a route to propose, prioritise and possibly test multi-omics network-based targets and repurposing opportunities.
